# Isolation and Identification of Dominant Bacteria From Black Soldier Fly Larvae (*Hermetia illucens*) Envisaging Practical Applications

**DOI:** 10.3389/fmicb.2021.665546

**Published:** 2021-05-13

**Authors:** Ellen Gorrens, Laurence Van Moll, Lotte Frooninckx, Jeroen De Smet, Leen Van Campenhout

**Affiliations:** ^1^Department of Microbial and Molecular Systems (M^2^S), Lab4Food, KU Leuven, Geel, Belgium; ^2^Leuven Food Science and Nutrition Research Centre (LFoRCe), KU Leuven, Leuven, Belgium; ^3^Laboratory for Microbiology, Parasitology and Hygiene (LMPH), Faculty of Pharmaceutical, Biomedical and Veterinary Sciences, University of Antwerp, Antwerp, Belgium; ^4^Thomas More University of Applied Sciences, RADIUS, Geel, Belgium

**Keywords:** black soldier fly, microbiome, fiber rich diets, isolates, culturable gut community

## Abstract

This study aimed to establish a representative strain collection of dominant aerobic bacteria from black soldier fly larvae (*Hermetia illucens*, BSFL). The larvae were fed either chicken feed or fiber-rich substrates to obtain a collection of BSFL-associated microorganisms. Via an approach based on only considering the highest serial dilutions of BSFL extract (to select for the most abundant strains), a total of 172 bacteria were isolated. Identification of these isolates revealed that all bacteria belonged to either the Proteobacteria (66.3%), the Firmicutes (30.2%), the Bacteroidetes (2.9%) or the Actinobacteria (0.6%). Twelve genera were collected, with the most abundantly present ones (i.e., minimally present in at least three rearing cycles) being *Enterococcus* (29.1%), *Escherichia* (22.1%), *Klebsiella* (19.8%), *Providencia* (11.6%), *Enterobacter* (7.6%), and *Morganella* (4.1%). Our collection of dominant bacteria reflects largely the bacterial profiles of BSFL already described in literature with respect to the most important phyla and genera in the gut, but some differences can be noticed depending on substrate, biotic and abiotic factors. Furthermore, this bacterial collection will be the starting point to improve *in vitro* digestion models for BSFL, to develop mock communities and to find symbionts that can be added during rearing cycles to enhance the larval performances, after functional characterization of the isolates, for instance with respect to enzymatic potential.

## Introduction

The past decade has seen the rapid expansion of the industrial insect rearing sector in response to the global increasing demand in high-quality protein for human consumption (Van Huis, 2016). One of the economically most important and most promising farmed insect species is the black soldier fly (*Hermetia illucens*; Diptera: Stratiomyidae). The larvae of the black soldier fly (BSFL) have attracted the attention of many researchers during recent years as they are able to convert low value biomass into valuable insects mass (Wang and Shelomi, 2017; Gold et al., 2018). BSFL may offer sustainable alternatives as raw materials for feed formulations and as source for biomaterials (Barragan-Fonseca et al., 2017; Almeida et al., 2020). Insects generally harbor diverse microorganisms in the gut which play pivotal roles in diverse aspects of insect physiology (Jang and Kikuchi, 2020). As a result, the gut microbial dynamics of these larvae are of specific interest to obtain insight in the insect’s physiology, and further, to increase the yield of the rearing or conversion process. In recent years, the literature available on the composition of the BSFL gut microbiota, possibly in relation with its substrate and other rearing factors, increased substantially, with examples of relevant studies including those of Jeon et al. (2011); Zheng et al. (2013), Wynants et al. (2018); Jiang et al. (2019), Ao et al. (2020); Klammsteiner et al. (2020), Liu et al. (2020); Raimondi et al. (2020) and Shelomi et al. (2020). Based on the aforementioned studies, it can be concluded that a set of genera regularly occurs in the larval gut, such as *Enterococcus*, *Providencia*, *Morganella*, and *Dysgonomonas*, although relative abundances vary among and within studies. Whereas the studies mentioned have focused on the identification of members of the BSFL microbial community, information on the functions of these persistent microorganisms is still scarce.

Most of those studies on the BSFL microbiome involve culture-independent techniques (such as metataxonomics based on 16S rRNA gene sequencing) to obtain a complete view on the composition of the microbial community. However, culture-dependent methods, involving incubation, isolation and identification steps, are still necessary to obtain pure cultures needed for research purposes and for industrial applications (Prakash et al., 2013). A collection of BSFL-associated microorganisms could be useful to gain knowledge about their specific functions. In that regard, Callegari et al. (2020) obtained a bacterial collection of microorganisms from the larval gut via plating in selective liquid and solid media in order to seek for key bacteria. The authors studied the hydrolytic profiles of their isolates and selected and tested possible candidates for bacterial administration trials. In their approach, the authors focused on obtaining isolates with specific functions. However, they did not include special measures to focus on the most dominant bacteria present, which is an element that is added in our study in order to obtain a collection of dominant BSFL-associated bacteria. When considering future industrial applications, it can be important to focus on dominant strains. It is reasonable to assume that a dominant strain is more likely to colonize and function in an *in vitro* environment mimicking the BSFL gut, or the real BSFL gut, than a strain that is hardly encountered in the ecological niche of the BSFL gut.

This study aimed to establish a representative culture collection of dominant aerobic BSFL-associated microbes envisaging practical applications. Some studies suggest that the rearing substrate is an important factor shaping the gut microbiota in BSFL (Jeon et al., 2011; Bruno et al., 2019b). Therefore, we established the collection by rearing BSFL on several substrate compositions. Keeping in mind the role BSFL could play in the circular bioeconomy as a converter of organic waste streams, our focus with respect to substrate composition was on the fiber fraction. Soluble carbon sources, such as soluble starch, were not included in the study, since BSFL can produce amylase enzymes when fed substrates containing starch (Kim et al., 2011; Bonelli et al., 2019; Bruno et al., 2019a). Since bacteria that are more adapted to digest a specific compound can be expected to have a higher fitness on specific diets (De Smet et al., 2018; Galassi et al., 2021), we hypothesize that these bacteria will be more abundant in the community and concomitantly are more likely to be isolated. We selected five fiber types to enrich (each individually) substrates with. Cellulose, hemicellulose and pectin are important non-starch carbohydrates in plant material, lignin is a recalcitrant fiber in plant material as well, and in addition we included keratin as a protein fiber. These components are found in various waste streams that can serve as rearing substrate, such as (i) animal manure (Rehman et al., 2017; Masih-Das and Tao, 2018), rice straw (Zheng et al., 2012) and maize straw (Gao et al., 2019) for cellulose, hemicellulose and lignin, (ii) fruit mixtures (Meneguz et al., 2018) for pectin, and (iii) slaughterhouse waste containing discarded body parts (Gold et al., 2020a) for keratin. These substrates have a complex and variable composition, and to ensure the repeatability of our study, we preferred to use mixtures of chicken feed with the individual and (more) pure fibers for rearing the larvae, rather than rearing them on the complex and unstandardized waste streams. Isolation of microorganisms was only focused on the higher serial dilutions obtained after BSFL extraction, aiming to isolate only those strains that are abundantly present in the gut of larvae reared on the substrates used. Finally, the focus is laid on aerobic bacteria, since anaerobic bacteria would be less easy to cultivate for future industrial applications.

## Materials and Methods

### Rearing and Sampling of Black Soldier Fly Larvae

**FIGURE 1:**
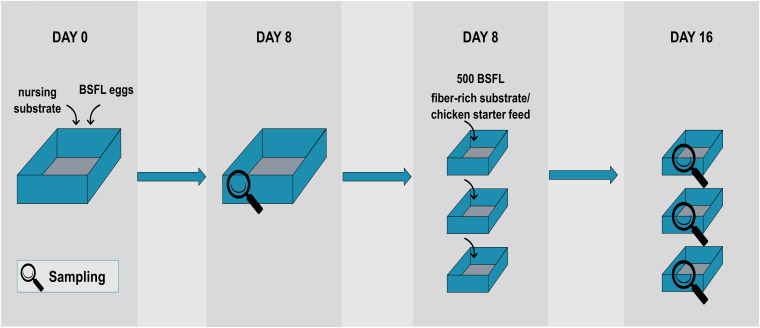
Schematic overview of the rearing protocol used, including the sampling points.

The BSFL used in this study were obtained from a colony reared at laboratory scale (Thomas More University College, Geel, Belgium). Figure 1 represents the rearing protocol used. Every cycle started at day 0 by placing 1 g of BSF eggs into a small tray placed on 200 g of nursing substrate, i.e., chicken starter feed (Startmeel voor Kuikens 259, AVEVE, Leuven, Belgium) and tap water [in an 1:1 ratio (w/v)]. The nursing substrate had a moisture content of approximately 50–60%. After 4 days, unhatched eggs were removed to ensure uniformity in larval age. At that time, the BSFL were transferred to another box (29 cm × 18 cm) to which 600 g nursing substrate [chicken starter feed and tap water in a 60:40 ratio (w/v)] was added. After 8 days, a first sample of larvae was taken and the larvae were divided over three separate containers (10 cm × 15 cm). Each box contained 100 g of new substrate (one of the fiber-rich types as described below) or chicken starter feed (=control) and approximately 500 BSFL (as determined by the average weight of three times 100 larvae), so that the density was 3.3 larvae/cm^2^. From then on, 100 g substrate was administered to the larvae every 2 days until day 16. All containers were placed in a climate chamber (Pharma 600, Weiss Technik, Belgium) under controlled environmental conditions (T = 27°C, RH = 60%).

The diets given from day 8 onward were supplemented with fiber(-rich) ingredients, being either lignin (Alkaline, TCI Europe N.V., Belgium), cellulose (Alphacel, VWR International, Belgium), pectin (Pektin A, Carl Roth, Germany), feather meal rich in keratin (Pluvera, Belgium), or pre-cut straw (Stro Voorgesneden, Aveve, Belgium) as a source of hemicellulose. For each ingredient, one rearing cycle with a diet in a 70:30 ratio (w/w) of chicken feed and fiber(-rich) ingredient was conducted. In order to provide the larvae an even less digestible substrate toward a more pronounced digestion of the fiber-rich substrate, another rearing cycle was performed in a 30:70 ratio (w/w). Since the diets containing 70% pectin or pre-cut straw did not support larval growth, they were eliminated from the trial, and spelt hulls (ForFarmers, Belgium) were introduced as a source of hemicellulose (of which both ratios were executed as well). Chicken starter feed served as a control substrate and the rearing cycles using the control feed were carried out twice. The substrates were moisturized with tap water to achieve a moisture content of circa 60–70% in order to ensure efficient separation of larvae from the residue upon harvesting (Cheng et al., 2017).

### Isolation of Microorganisms

For each cycle, a sample was taken at day 8 before distributing the larvae over separate containers, and another sample was taken from each of the three boxes at day 16 (i.e., in total four samples per cycle). Each sample contained about 30 g of BSFL. To eliminate the microbiota present on the exoskeleton and in this way to focus on microorganisms present in the interior of the larvae, all samples were given a rinsing disinfection treatment as described by Wynants et al. (2018). In summary, the BSFL were first washed under running tap water and then subjected to three washing steps of 1 min at 200 rpm on a laboratory shaking table (Unimax 1010, Heidolph, Germany), first once with 70% ethanol and then twice with sterile distilled water. Subsequently, the insects were aseptically blended with a home type mixer (Bosch CNHR 25) to obtain a homogenous mixture. Tenfold serial dilutions were prepared and surface-plated on Plate Count Agar (PCA, Biokar Diagnostics, Beauvais, France). Plates obtained from dilutions starting from 10^–5^ until 10^–9^ were considered, since only highly abundant microorganisms are present in those dilutions and rare or less frequent microorganisms are not. All plates were incubated aerobically at 30°C for 2–3 days. From those plates, five to ten morphologically different colonies were picked and streaked onto new PCA plates in order to obtain pure cultures. Those plates were incubated at 30°C for 24–48 h. Next, one individual colony was picked from each plate and inoculated in Luria Bertani (LB) liquid broth (10.0 g/l peptone (Biokar Diagnostics, France), 5.0 g/l yeast extract (VWR, Belgium), 5.0 g/l NaCl), and grown overnight at 30°C on a shaker (Orbital mini shaker, VWR, Belgium) at 150 rpm. Visibly turbid cultures were maintained as 25% glycerol stock at −21 and −80°C.

### Identification of Isolates

By picking up colonies from plates in order to obtain isolates, it is possible that a single strain was collected more than once. To find out whether a strain occurred more than once in the collection, Random Amplification of Polymorphic DNA (RAPD) was performed to identify multiple isolates originating from the same strain. Then, unique strains were subjected to Matrix Assisted Laser Desorption Ionization Time-Of-Flight (MALDI-TOF) mass spectrometry for identification.

For the RAPD analysis, each isolate from the stock culture collection was grown overnight on PCA. From each plate, one individual colony was suspended in 20 μl milli-Q water. RAPD was performed using suspended cells (i.e., no DNA extraction as described by Hilton et al., 1997) and using the universal primer GTG_5_ (5′-GTG GTG GTG GTG GTG-3′). PCR was carried out in 30 μl reaction volumes containing 2 μl of PCR template, 0.7 μl primer (20 μM, IDT, Belgium), 0.1 μl DreamTaq polymerase (5 U/μl, Life Technologies, Belgium), 0.6 μl 10 nM deoxynucleotide triphosphates (dNTP’s) (Thermo Fisher Scientific, Belgium), 3 μl 10 × DreamTaq Green buffer (Thermo Fisher Scientific, Belgium), and 23.6 μl milli-Q water. A negative control for which the DNA was replaced by sterile milli-Q water was included in each PCR run. After initial denaturation for 10 min at 95°C, each PCR reaction comprised 10 cycles of denaturation (45 s, 95°C), annealing (45 s, 36°C), and extension (2 min, 75°C), followed by 25 additional cycles where the annealing temperature was set at 50°C. Subsequently, post-cycle elongation was performed for 5 min at 72°C. The resulting PCR products were visualized on an 1.5% agarose gel. Identification of band fragments and comparison of the different patterns were performed using GelAnalyzer (Version 19.1 for Windows). Isolates with the same RAPD-profile were combined into the same group.

Of each group, at least one isolate was identified using MALDI-TOF mass spectrometry (Lavetan, Turnhout, Belgium). A Bacterial Test Standard (BTS), being *Escherichia coli*, and the strain *Salmonella enterica* serovar *Infantis* (LMG 18746) were included as positive control. The obtained spectrum was compared against a spectra database, i.e., MBT Compass Library (Bruker, United States of America) containing 7,854 reference spectra of 2,748 different microorganisms. The match of the obtained spectrum with spectra from this library was expressed as a log-score between 0.00 and 3.00. For accurate species identification, a log-score of minimum 2.00 is required (de Almeida Júnior et al., 2014). Isolates with a log-score below 2.00 were therefore identified via amplification and sequencing of the bacterial 16S ribosomal RNA region. Briefly, cells were dissolved in 20 μl ultra-pure water and the DNA was set free via boiling lysis (10 min at 95°C). The 16S rRNA gene was amplified using the primers 27F (5′-AGA GTT TGA TCM TGG CTC AG-3′) and 1492R (5′-CTA CGG CTA CCT TGT TAC GA-3′) (Mapelli et al., 2013; Soldan et al., 2019). PCR fragments were partially sequenced at Eurofins Genomics using both primers. The obtained sequences were assembled using ContigExpress (Vector NTI, Invitrogen) and then aligned against the EzBioCloud database (Yoon et al., 2017).

## Results

### Identification of the Isolates

**FIGURE 2:**
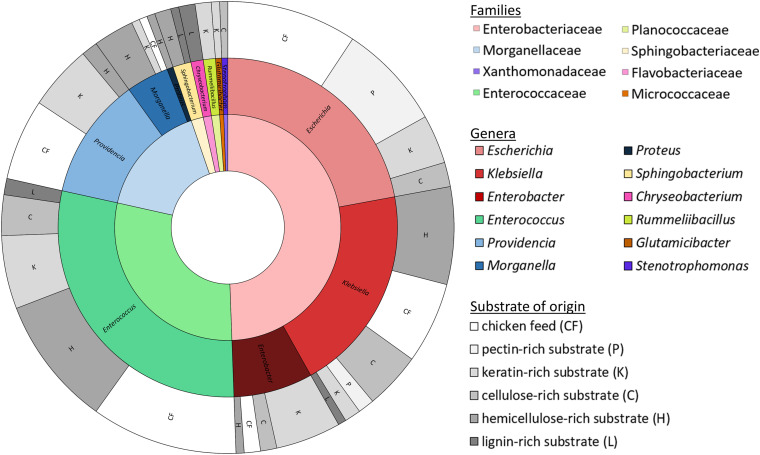
Families (inner circle, cf. legend on the right side) and genera (middle circle, cf. legend on the right side) of the 172 identified isolates with the substrate of origin (outer circle; cf. legend on the right side).

Isolates were obtained from all fiber-rich substrates as well as the chicken feed. In total, a collection of 172 isolates was established. These isolates were ordered in 31 groups according to their RAPD-profile. Figure 2 gives an overview of the families and genera of these organisms, which all appeared to be bacteria, and the substrate they were isolated from. All the isolates belonged to one of four phyla, being the Proteobacteria (66.3%), the Firmicutes (30.2%), the Bacteroidetes (2.9%) and the Actinobacteria (0.6%). The largest group, the Proteobacteria, encompassed the families of the Enterobacteriaceae (49.4%), the Morganellaceae (16.3%), and the Xanthomonadaceae (0.6%). The Firmicutes covered some Enterococcaceae (29.1%) and Planococcaceae (1.2%). Bacteroidetes were assigned to Sphingobacteriaceae (1.7%) and Flavobacteriaceae (1.2%). The Actinobacteria were only represented by one family, being the Micrococcaceae (0.6%).

These four phyla covered together twelve different genera. The Enterobacteriaceae were represented by the genera *Escherichia* (22.1%), *Klebsiella* (19.8%) and *Enterobacter* (7.6%). *Enterococcus* was the only genus found in the family of the Enterococcaceae, but it accounted for 29.1% of all isolates. *Providencia* (11.6%), *Morganella* (4.1%) and *Proteus* (0.6%) were the genera present from the family of the Morganellaceae. Those three families together were responsible for 94.8% of the collection. Further, isolates from the genus *Sphingobacterium* (1.7%), belonging to the Sphingobacteriaceae, *Chryseobacterium* (1.2%) from the Flavobacteriaceae, *Rummeliibacillus* (1.2%) from the Planococcaceae, *Glutamicibacter* (0.6%) from the Micrococcaceae and *Stenotrophomonas* (0.6%) from the Xanthomonadaceae were also picked up.

### Distribution of the Isolates on the Different Substrates

Fiber-rich substrates containing either cellulose, hemicellulose, keratin, lignin or pectin in different ratios with chicken feed (70 and 30%), and chicken feed as such were administered to BSFL in different rearing cycles. During these cycles, bacteria were isolated on day 8 for chicken feed and on day 16 for chicken feed and the fiber-rich substrates. The majority of the isolates originated from the control group (*n* = 57 or 33.1%), the hemicellulose-rich diet (*n* = 39 or 22.7%), and the keratin-rich diet (*n* = 37 or 21.5%). A smaller fraction was collected from the cellulose-rich (*n* = 18 or 10.5%), the pectin-rich (*n* = 15 or 8.7%) and the lignin-rich substrate (*n* = 6 or 3.5%).

**FIGURE 3:**
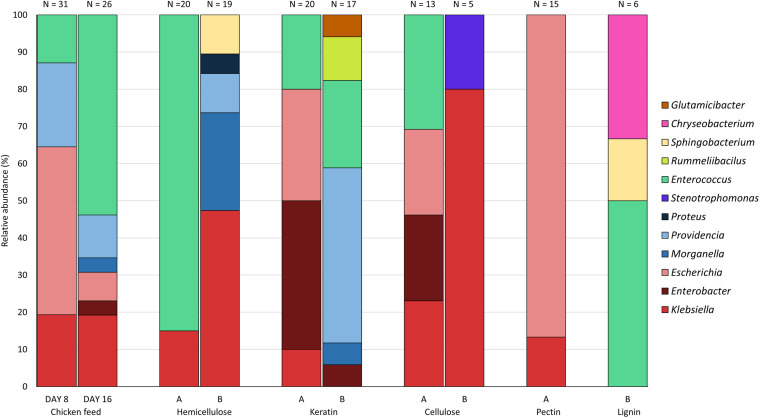
Relative abundance of the genera isolated from black soldier fly larvae fed on chicken feed and various fiber-rich diets (A = 70% chicken feed and 30% fiber; B = 30% chicken feed and 70% fiber). Isolates from larvae fed on chicken feed were obtained at days 8 and 16, whereas isolates from fiber-rich substrate were only obtained at day 16. N is the number of isolates obtained from larvae fed on the specific fiber-rich substrate.

Figure 3 presents the relative abundance of the genera of the isolates found for each substrate. All genera of the three most abundant families found in this study, i.e., the Enterobacteriaceae, the Enterococcaceae, and the Morganellaceae, were discovered on chicken starter feed, with the exception of *Proteus*. Comparison of day 8 (*n* = 31) and day 16 (*n* = 26) shows that the obtained patterns showed some similarity, with *Klebsiella*, *Escherichia*, *Providencia* and *Enterococcus* being the genera occurring the most. On day 16, the genera *Morganella* and *Enterobacter* were isolated as well. For the 30% hemicellulose diet (*n* = 20), the only genera discovered were *Klebsiella* and *Enterococcus*, with the latter one accounting for 85% of all isolates collected from this substrate. Interestingly, no *Enterococcus* was isolated from larvae fed with substrate containing 70% hemicellulose (*n* = 19), yet *Klebsiella*, *Morganella*, *Providencia*, *Sphingobacterium* and *Proteus* were isolated. In the BSFL fed with 30% keratin *Klebsiella*, *Enterobacter*, *Escherichia*, and *Enterococcus* were present as abundant (and culturable) genera (*n* = 20). Bacteria isolated from larvae fed on the other substrate, being 70% keratin (*n* = 17), showed a high diversity. In addition to genera of the three most abundant families, *Rummeliibacillus* and *Glutamicibacter* were present in the larval interior as well. The relative abundances of the bacteria obtained from BSFL fed with 30% cellulose (*n* = 13) were quite similar to those of 30% keratin, whereas bacteria isolated from larvae fed on 70% cellulose only belonged to the genera *Klebsiella* and *Stenotrophomonas*. Nevertheless, it is important to note that only five isolates were recovered on 70% cellulose. Interestingly, no members of Morganellaceae were detected in both rearing cycles of cellulose-rich substrates. Rearing of BSFL on pectin-rich substrate has only been performed on 30% pectin (*n* = 15), as indicated earlier, and 87.0% of the isolates were from *Escherichia*. For the lignin-based diet containing 30% lignin, no isolates were obtained using the protocol described in this study. In another study in our group and connected to this work, using selective plates and not just considering the highest dilutions, isolates were obtained, but these data are still to be published. For the lignin-based diet containing 70% lignin, only six isolates were recovered, and they belonged to three genera, being *Enterococcus*, *Sphingobacterium* and *Chryseobacterium*.

From all genera found in this study, *Klebsiella* and *Enterococcus* were present in most of the rearing cycles (Figure 3), indicating a high general abundance of these genera in the gut of BSFL, i.e., an abundance which is independent from the diet. In contrast, *Rummeliibacillus*, *Glutamicibacter*, *Chryseobacterium*, *Proteus*, *Stenotrophomonas* and *Sphingobacterium* were found in only one or two rearing cycles. Identification on species level is given in Supplementary Table 1. For isolates identified via amplification of the 16S rRNA gene, the top-hit taxon is given.

## Discussion

### Representativeness of the Collection

The microbiome of BSFL has been described in several recent studies. In order to enable the comparison of our results with those already available, an overview of the literature covering the BSFL microbiome composition is given in Table 1. An important distinction has to be made between research using culture-independent methods (such as metataxonomics based on 16S rRNA gene sequencing) and research, such as ours, where the culturable fraction is investigated. Culture-independent methods may yield a more comprehensive and complete view on the composition of the microbial community, since also Viable But Non-Culturable (VBNC) strains are included (Oliver, 1993). However, strains usable for additional research and industrial applications need to be culturable. Hence, an approach based on isolation and culturing strains still plays a crucial role (Prakash et al., 2013). Further, it can be noted that plates were not incubated anaerobically and that part of the microbiota, containing the strictly anaerobic organisms, is missed in this way. Strictly anaerobic bacteria were not considered in this study, due to the applications envisioned for the collection. Organisms that would have to be cultured in a strictly anaerobic way would be difficult to handle in large scale applications and would involve additional costs over (facultatively) aerobic ones.

**TABLE 1:** Summary of literature data (from 2011 onward) involving an assessment of the composition of the microbiota of BSFL, either in a culture-dependent (D) or in a culture-independent (I) way.

		**Main genera^*a*^**																			
**Study authors and year of publication**	**D/I**	*Actinomyces*	*Bacillus*	*Bacteroides*	*Campylobacter*	*Clostridium*	*Corynebacterium*	*Dysgonomonas*	***Enterococcus***	*Ignatzschineria*	***Klebsiella***	*Lactobacillus*	*Miniimonas*	***Morganella***	*Parabacteroides*	***Proteus***	***Providencia***	*Pseudomonas*	***Sphingobacterium***	***Stenotrophomonas***	*Vagococcus*
Jeon et al. (2011)	I and D								X		X			X		X					
Zheng et al. (2013)	I			X				X									X		X	X	
Cai et al. (2018)^*b*^	I	X							X	X	X					X	X				
Wynants et al. (2018)	I								X					X			X	X			
Bruno et al. (2019b)^*c*^	I					X	X	X	X					X		X	X	X	X		X
Jiang et al. (2019)^*d*^	I		X					X	X			X									
Ao et al. (2020)	I	X				X			X	X	X			X		X	X				X
Callegari et al. (2020)	D								X		X			X			X	X		X	
Cifuentes et al. (2020)	I	X	X			X		X	X		X			X			X			X	
Khamis et al. (2020)	I					X		X	X	X		X		X			X				
Klammsteiner et al. (2020)	I	X						X	X					X							
Raimondi et al. (2020)	I		X								X						X				
Shelomi et al. (2020)	I and D							X							X						
Wu et al. (2020)^*b*^	I				X										X						
Galassi et al. (2021)	I				X		X	X	X		X			X			X		X		
Liu et al. (2021)	I							X					X	X							
Wu et al. (2021)^*b*^	I			X	X			X					X								

*Studies were found via Pubmed using the following search terms: Hermetia illucens, black soldier fly larvae, microbiota, bacterial community, bacterial diversity and gut. Studies are presented in the order of online publication. Genera that are considered as main genera in at least two studies are given. Genera that are isolated in our study are in bold. ^a^Main genera are genera that were described as “core/common members” of the larval gut, and/or were omnipresent (supplementary information not included) in all rearing cycles or life stages, and/or were found with a relative abundance of minimum 6%. ^b^Only genera of the initial group (no addition of tetracycline or heavy metals) were considered. ^c^Based on supplementary data. ^d^Based on a figure in the paper.*

Most authors of recent studies dissected the larval midgut in order to get more precise information on the intestinal microbiota, but due to the labor-intensive step of dissecting, this approach reduces the number of larval guts on which their results are based. For instance, Shelomi et al. (2020) only examined three larval guts per rearing cycle. Therefore, it was decided to use a more representative number of larvae in this study, to increase the relevance of the collection as a representation of the intestinal microbiota. For this reason, the larval midgut was not dissected, but larvae were rather disinfected to remove microorganisms at the external surfaces. The drawback of this approach, however, is that it cannot be claimed in our study that it focusses on the gut microbiome only. Indeed, it cannot be excluded that other organs or tissues in the BSFL interior also contain microorganisms, but this is largely unexplored.

It can be observed in Table 1 that most studies use culture-independent methodologies. Even though only a minor fraction of a community unveiled by sequencing methods is culturable (Alain and Querellou, 2009; Prakash et al., 2013), the bacterial profile found within these culture-independent studies is to some extent comparable to that found via our approach. Overall, these studies reported that the most representative phyla are the Proteobacteria, the Firmicutes, the Bacteroidetes and to a lesser extent the Actinobacteria (Jeon et al., 2011; Zheng et al., 2013; Wynants et al., 2018; Bruno et al., 2019b; Jiang et al., 2019; Zhan et al., 2019; Ao et al., 2020; Klammsteiner et al., 2020; Liu et al., 2020, 2021; Raimondi et al., 2020; Shelomi et al., 2020; Galassi et al., 2021; Wu et al., 2021). In our collection, these four phyla are also represented: the Proteobacteria (66.3%), the Firmicutes (30.2%), the Bacteroidetes (2.9%) and the Actinobacteria (0.6%). Within these phyla, isolates from twelve genera were collected, with the dominant ones being *Enterococcus* (29.1%), *Escherichia* (22.1%), *Klebsiella* (19.8%), *Providencia* (11.6%), *Enterobacter* (7.6%), and *Morganella* (4.1%). Interestingly, these genera can also be found frequently and abundantly in culture-independent studies as well. More specifically, the genus *Enterococcus*, isolated the most in our study, has also been found in most studies shown in Table 1, indicating an occurrence independently of substrate and other factors. The same conclusions can be drawn for the genera *Providencia* and *Morganella*. Interestingly, *Enterococcus* sp., *Morganella* sp., and *Providencia* sp. were all found to be omnipresent in three industrial and four laboratory rearing cycles in the study of Wynants et al. (2018) and in three sets of larval groups fed on different diets in the study of Jeon et al. (2011). In addition, *Providencia* spp. have been detected throughout all life stages of BSF (Zheng et al., 2013), indicating that the genus is transmitted in a vertical way, i.e., from parents to offspring over different cycles. Even though not mentioned often in Table 1, the genera *Klebsiella* (Jeon et al., 2011; Ao et al., 2020; Callegari et al., 2020; Cifuentes et al., 2020), *Escherichia* (Jeon et al., 2011; Callegari et al., 2020), and *Enterobacter* (Callegari et al., 2020; Cifuentes et al., 2020) were found in the interior of BSFL as well. Some genera, being *Sphingobacterium* (1.7%), *Rummeliibacillus* (1.2%), *Chryseobacterium* (1.2%), *Glutamicibacter* (0.6%), *Proteus* (0.6%), and *Stenotrophomonas* (0.6%), were only discovered once or twice in this study. To the author’s knowledge, it is the first time that the genera *Chryseobacterium* and *Glutamicibacter* are linked to the interior of BSFL, whereas the other aforementioned isolates have been identified in BSFL previously. For example, the genera *Sphingobacterium* (Zheng et al., 2013; Wynants et al., 2018; Bruno et al., 2019b), *Rummeliibacillus* (Zheng et al., 2013), *Proteus* (Jeon et al., 2011; Zheng et al., 2013; Ao et al., 2020; Cifuentes et al., 2020; Khamis et al., 2020; Raimondi et al., 2020) and *Stenotrophomonas* (Zheng et al., 2013; Callegari et al., 2020; Cifuentes et al., 2020) have been recovered in larvae.

Until now, only one other in-depth study on the culturable section of the BSFL gut has been performed (Callegari et al., 2020). In that study, the larval guts were dissected, but their collection is based on only four larval guts whereas in this study, a much larger (and likely more representative) number of larvae were used. In addition, Callegari et al. plated on enriched and selective media in search of bacteria with specific functions. Although the authors investigated the culturable fraction of the larval gut in a specific and different manner, their bacterial collection showed some similarities to ours. In fact, all the dominant genera described above were isolated, just as *Proteus* and *Stenotrophomonas*, but they found other genera, such as *Acinetobacter*, *Pseudomonas*, and *Micrococcus*, associated with BSFL as well.

Although many resemblances are noted between bacterial profiles of BSFL described in literature and our isolates, there are still some notable differences. For instance, *Dysgonomonas* spp. were not recovered in our study, whereas the genus has been found frequently in other work (Zheng et al., 2013; Cai et al., 2018; Wynants et al., 2018; Jiang et al., 2019; Zhan et al., 2019; Ao et al., 2020; Cifuentes et al., 2020; Khamis et al., 2020; Klammsteiner et al., 2020; Shelomi et al., 2020), even as main members of the gut community (Zheng et al., 2013; Cai et al., 2018; Cifuentes et al., 2020; Klammsteiner et al., 2020; Shelomi et al., 2020). The same applies to the genera *Lactobacillus*, *Acinetobacter*, and *Bacillus* (Wynants et al., 2018; Jiang et al., 2019; Callegari et al., 2020; Cifuentes et al., 2020; Khamis et al., 2020; Raimondi et al., 2020). This may be attributed to differences in the culture-independent and -dependent approach, as pointed out earlier.

### Existence of a Core Microbiome

A predominant core community has often been identified in different insect species, affecting the health and survival of the insect itself (Engel and Moran, 2013). As to BSFL, there is no general consensus on whether they contain a core microbiota and if so, which members belong to it. In our study, most of the larval groups fed on different diets share several genera, yet in other proportions. Genera that were found in at least three rearing cycles include *Enterococcus*, *Escherichia*, *Klebsiella*, *Providencia*, *Enterobacter*, and *Morganella*. As stated earlier, these genera are frequently found in the larval interior and might thus be regarded as core members of the gut community. Indeed, most studies described previously in Table 1 have linked a recurring set of dominant genera to the gut of *H*. *illucens*, independently of rearing procedures. Most of these presumed core microbiomes include at least one of the genera *Enterococcus*, *Dysgonomonas*, *Providencia*, *Morganella*, *Klebsiella*, *Enterobacter*, and/or *Actinomyces*, yet with varying abundances (Jeon et al., 2011; Wynants et al., 2018; Ao et al., 2020; Cifuentes et al., 2020; Klammsteiner et al., 2020; Shelomi et al., 2020). These results are in agreement with our findings that those genera are possibly persistent regardless of the feed used. Interestingly, the composition of those presumed core microbiomes differs between studies in terms of number of genera and their abundancies. For instance, *Dysgonomonas*, *Porhyromonas*, and *Parabacteroides* have been assigned to the gut core microbiome (Shelomi et al., 2020), whereas in another study, none of these genera were considered to be a part of it (Ao et al., 2020). Due to these differences, it has been suggested that core bacterial microbiomes of *H*. *illucens* vary with location and feed (De Smet et al., 2018; Shelomi et al., 2020). Furthermore, the gut microbiota might be influenced by a so-called “house flora,” i.e., species typically present in the rearing environment (crates, production site, equipment, …) (Wynants et al., 2018), but to the author’s knowledge, no reports exist that document the house flora of BSFL production facilities.

In line with this discussion, the influences of the substrate, biotic [such as BSF strain (Khamis et al., 2020)] and abiotic factors (such as temperature) on shaping the gut microbiota are not clear yet. In this respect, so far substrate has been investigated the most as possible influencing factor. For instance, Bruno et al. (2019b) investigated the effect of different substrates on the microbiota of BSFL, including the different regions of the midgut. They concluded that the substrate played a major role in the composition of the microbiota. In another study, the least complex diet, i.e., cooked rice, resulted in the least diverse intestinal microflora, followed by calf forage and food waste (Jeon et al., 2011). On the other hand, no clear overlap between the community composition of the substrates and that of the BSFL fed on it was observed in other studies (Wynants et al., 2018; Cifuentes et al., 2020; Shelomi et al., 2020), suggesting that diet does not play a key role in shaping the BSFL’s gut microbiome. In addition, the effect of consecutive rearing cycles and inter-batch variability should still be deciphered, as highlighted by Wynants et al. (2018). Further, environmental conditions, such as temperature (Raimondi et al., 2020), can also influence the microbiota composition. Additionally, very little is currently known about the impact of the different regions of the larval midgut on the microbiome of BSFL. A higher bacterial load, yet coinciding with a lower microbial diversity, was noticed in the posterior tract compared to the anterior region, suggesting that the bacterial community becomes less diverse along the digestive tract and consequently that the core microbiome is site-dependent (Bruno et al., 2019b). Unraveling these factors will add relevant knowledge about the bacterial microbiota of BSFL. Based on our results and those of others, there is an indication that the gut of BSFL shows common phyla and genera as the main constituents in the gut (considered over its complete length), but some variation on this core can occur depending on feed, location, and possible other factors (such as BSF strain).

### Possible Applications of the Culturable Gut Bacteria

The collection of isolates obtained offers an interesting basis for research and industrial applications. Applying the potential of these isolates in different fields might lead to promising innovations. For our established collection of 172 bacteria, we consider three developmental prospects.

The first development is situated at research level. A current challenge in the emerging insect sector is the fact that process performance can vary considerably when administering different biowaste streams to BSFL (Gold et al., 2018). Gold et al. (2020b) attempted to asses in a direct and cost-effective way the possibility of various biowastes to be processed by BSFL by predicting the biowaste conversion performance using *in vitro* digestion models. In these models, three digestion phases are distinguished and in each phase the diet is diluted with a digestion fluid and incubated for a specific time. The pH of the digestion fluid is adjusted and various enzymes are added during the digestion steps (Brodkorb et al., 2019; Gold et al., 2020b). Comparison of their *in vitro* and *in vivo* results showed that the model was able to broadly distinguish between the best and the worst performing diets, but several major discrepancies were observed as well. Adding microorganisms in the digestion step to simulate the midgut microbiota may offer a next step in optimalization of *in vitro* models, covering a broader range of biological processes taking place in the gut than in the current model. For instance, microbially produced enzymes may partially digest certain components present in the feed (Gold et al., 2020b). Since our collection was established from the interior—and presumably mainly the gut—of BSFL and our isolates indeed reflect the microbial communities of the BSFL gut described in literature, they can be used as inoculum for *in vitro* digestion models for BSFL and thus to increase the reliability of the model in a standardized and repeatable way, creating a strong tool for future research.

Further, next generation sequencing (NGS) has been intensively used in recent studies to increase our understanding of BSFL microbial communities. During sequencing, in addition to a negative control, a positive control can be used, consisting of a known collection of microbial strains, or a known quantity of DNA from multiple microorganisms in known abundances. Such positive control is called a “mock community”. Adding a mock community to a sequencing run can validate whether the expected abundances are recovered, and thus it can improve the quality of the sequencing results (Nguyen et al., 2015). A diverse mock community is preferred, meaning that it consists of a wide range of taxonomic groups (Flynn et al., 2015; Nguyen et al., 2015). Recently, a mock community comprised of representatives of 374 insect species has been assembled (Braukmann et al., 2019). Since our collection consists of the main phyla and genera associated with *H*. *illucens*, a proper mock community for culture-independent studies on BSFL can now be designed, as is subject of our ongoing research.

Finally, microorganisms could be administered during industrial rearing cycles as symbionts in order to boost the larval performances on difficult to digest substrates. Indeed, some rearing substrates, such as dairy manure (Rehman et al., 2017) and rice straw (Zheng et al., 2012; Manurung et al., 2016), are hard to valorize by BSFL due to their high cellulose, hemicellulose and/or lignin content. If insect rearing facilities could successfully feed these difficult to digest streams to their larvae, production costs of the larvae could be lower (due to a lower feed cost) and at the same time insect production could take even a better position in a circular economy. In the development of this application, it is necessary to investigate the properties with respect to the enzyme production of the isolates. This presents another part of our ongoing research on the collection of BSFL isolates obtained. Several examples can be found in literature of successful application of bacteria in BSFL rearing, where enzyme production is considered to be (at least one) of the mechanisms by which the added cultures support growth of and/or conversion by BSFL. In this way, Jiang et al. (2019) demonstrated the importance of certain gut bacteria in BSFL during vermicomposting: *Enterococcus* and *Providencia* (also isolated in our study) were abundantly present in the process. The authors suggested that their influence might be related to carbohydrate-active enzymes they produce, and to the hydrogen, nitrogen and sulfur metabolisms they possess. In another study, the presence of *Providencia* has been strongly correlated with the total nitrogen content of manure fed to BSFL, but also with the crude fat content. Hence, *Providencia* was suggested playing a role in protein and lipid conversion in the gut (Ao et al., 2020) and this hypothesis was strengthened by the study of Callegari et al. (2020). Here, most bacteria that were isolated from the BSFL gut and that were able to degrade uric acid, belonged to the genus *Providencia*. In addition, pectinolytic activity has been related to *Klebsiella* spp. and *Stenotrophomonas* spp. strains associated with BSFL (Callegari et al., 2020). *Klebsiella* spp. were also found in larvae fed the pectin-rich diet in our study, but not exclusively in larvae fed on this type of diet. That may indicate that *Klebsiella* spp. can fulfill other functional roles in the gut of BSFL as well. It has already been proven repeatedly that the addition of bacteria during rearing of BSFL can increase larval performance (Yu et al., 2011; Rehman et al., 2019; Somroo et al., 2019; Callegari et al., 2020). The addition of *Bacillus licheniformis* HI169 has resulted in positive enhancement of larval growth (Callegari et al., 2020). Indeed, that strain has different hydrolytic activities, such as degradation of cellulose, starch and uric acid. Since our larvae were challenged with difficult-to-digest substrates, it can be hypothesized that bacteria with functional properties had a competitive advantage compared to other microorganisms (De Smet et al., 2018) and might thus lead to promising results regarding larval performance. Less frequently occurring isolates from our collection were mainly found in BSFL fed with the highest ratio of fiber-rich ingredient to chicken feed, indicating that these bacteria are more adapted to digest this type of fiber. It would thus be interesting to focus in future studies on these less frequently occurring isolates.

## Conclusion

A representative collection of dominant aerobic bacteria isolated from the interior of BSFL was obtained in this present study. Even though only a small fraction of a community unveiled by sequencing methods is culturable (Alain and Querellou, 2009; Prakash et al., 2013), this collection of 172 isolates was mainly constituted of members that have already been described to be frequently found in the interior of BSFL by a number of studies mainly using culture-independent strategies. This indicates that core members of the larval gut microbiota were isolated in this study and that the methodology of using only high dilutions during the isolation process indeed leads to the isolation of the dominant microorganisms. In total, twelve genera were collected with the main ones being *Enterococcus* (29.1%), *Escherichia* (22.1%), *Klebsiella* (19.8%), *Providencia* (11.6%), *Enterobacter* (7.6%), and *Morganella* (4.1%). Taking into account our work and that of others, the microbiota of BSFL seems to consist of common members, but their presence and abundance may vary depending on diet, biotic and abiotic factors. Still, the influence of factors such as the stages in rearing and the location in the gut needs more research.

The microorganisms isolated in this study are an interesting tool for various future research applications. First, *in vitro* digestion models for BSFL can be improved by the addition of isolates from our collection during the digestion step. Secondly, a mock community specifically designed for sequencing the microbiota of BSFL can be developed using our bacterial collection. Lastly, the collection potentially contains isolates that can be added as probiotic symbiont during rearing to enhance larval performance such as growth and/or biowaste conversion.

## Data Availability Statement

The original contributions presented in the study are included in the article/Supplementary Material, further inquiries can be directed to the corresponding author/s.

## Author Contributions

EG, LV, JD, and LV contributed to the conception and the design of the study. LF performed most work on rearing the insects, being assisted by EG. EG and LV performed the work on isolating bacterial strains. EG and JD performed the identification of the collection. EG wrote the first draft of the manuscript. LV wrote certain sections of the manuscript and did the proofreading of the first version. All authors contributed to manuscript revision, read and approved the submitted version.

## Conflict of Interest

The authors declare that the research was conducted in the absence of any commercial or financial relationships that could be construed as a potential conflict of interest.
